# Constitutive Expression of Pluripotency-Associated Genes in Mesodermal Progenitor Cells (MPCs)

**DOI:** 10.1371/journal.pone.0009861

**Published:** 2010-03-25

**Authors:** Simone Pacini, Vittoria Carnicelli, Luisa Trombi, Marina Montali, Rita Fazzi, Edoardo Lazzarini, Stefano Giannotti, Mario Petrini

**Affiliations:** 1 Hematology Division, Department of Oncology, Transplants and New Advances in Medicine, University of Pisa, Pisa, Italy; 2 Dipartimento di Scienze dell'Uomo e dell'Ambiente, University of Pisa, Pisa, Italy; 3 Dipartimento di Endocrinologia e Metabolismo, Ortopedia e Traumatologia, Medicina del Lavoro, University of Pisa, Pisa, Italy; National Institute on Aging, United States of America

## Abstract

**Background:**

We recently characterized a progenitor of mesodermal lineage (MPCs) from the human bone marrow of adults or umbilical cord blood. These cells are progenitors able to differentiate toward mesenchymal, endothelial and cardiomyogenic lineages. Here we present an extensive molecular characterization of MPCs, from bone marrow samples, including 39 genes involved in stem cell machinery, differentiation and cell cycle regulation.

**Methodology/Principal Findings:**

MPCs are cytofluorimetrically characterized and quantitative RT-PCR was performed to evaluate the gene expression profile, comparing it with MSCs and hESCs lines. Immunofluorescence and dot-blot analysis confirm qRT-PCR data. MPCs exhibit an increased expression of OCT4, NANOG, SALL4, FBX15, SPP1 and to a lesser extent c-MYC and KLF4, but lack LIN28 and SOX2. MPCs highly express SOX15.

**Conclusions/Significance:**

MPCs express many pluripotency-associated genes and show a peculiar *Oct-4* molecular circuit. Understanding this unique molecular mechanism could lead to identifying MPCs as feasible, long telomeres, target cells for reprogramming with no up-regulation of the p53 pathway. Furthermore MPCs are easily and inexpensively harvested from human bone marrow.

## Introduction

Pluripotency has been the object of increasing interest and has been extensively debated over the last few years. Adult stem cells provide fascinating prospects for clinical applications emerging from the recent advances in obtaining pluripotent cells. As clearly summarized by Beltrani *et al* [1], at present, pluripotent cells may be obtained by two main approaches: 1) inducing pluripotency in somatic cells by manipulation or 2) by identification and isolation, in adult tissues, of very rare cells exhibiting multilineage differentiation. Different strategies for reprogramming somatic cells along with different approaches in isolating and culturing rare adult stem cells, i.e. marrow-isolated adult multilineage inducible (MIAMI) [2], very small embryonic-like (VSEL) [3] and multipotent adult progenitor (MAPC) [4] cells, lead to controversial interpretation of the biological aspects of pluripotency. However, increased interest and a large number of studies on induced pluripotent stem cells (iPSCs) [5], [6] help to clarify some relevant aspects of stem cell machinery. The first evidence that forced the expression that four transcription factors (*Oct-4*, *Sox2*, *Klf-4* and *c-myc*) can induce pluripotency in mouse fibroblasts [7] started the investigation of relevant aspects about the molecular mechanisms that govern pluripotency. Subsequently, Yu *et al.* [8] obtained iPSCs in humans by transfecting with OCT4, SOX2, NANOG and LIN28. Many attempts have been carried out to reveal which factors are really required to obtain iPSCs and if transfection is a unique method. In order to reduce the risks of genetic modifications, representing the main obstacle for the safe clinical use of these cells, several groups have tried to obtain pluripotency by transient transfection [9], transposones [10], adenoviral [11], episomal vectors [12] or recombinant proteins [13]. Nonetheless, even though these new approaches lead to the preservation of the genome integrity of reprogrammed cells, current techniques to induce pluripotency are not at present pursuable due to their low efficiency and only partial reprogramming. Recent studies suggest that the use of adult progenitor cells, i.e. neural stem cells in mice [14] or hematopoietic stem cells [15], as starting cells give rise to iPSCs more rapidly and with a higher efficiency, probably being epigenetically responsive to nuclear resetting. Furthermore, the use of progenitors constitutively expressing one or more reprogramming factors should allow the obtaining of iPSCs with a reduced number of transfection vectors. Neural stem cells that constitutively express *Sox2* have been reprogrammed by the single factor OCT4 in mice [16] and humans [17]. From the assumption that progenitors are more reliable, due to the less stringent epigenetic restriction, Eminli *et al*, evaluating as starting cells hematopoietic stem cells versus mature T and B cells, provide evidence that the differentiation stage could influence the yield of iPSC colonies [15], according to the “elite” model of reprogramming [18].

Recently we described a simple and inexpensive culture method to isolate highly selected mesodermal progenitor cells (MPCs) from human bone marrow and umbilical cord blood [19], which may be useful as starting cells for reprogramming. These cells have been previously described in mesenchymal stromal cell (MSC) cultures, performed in autologous serum [20]. MPCs exhibited unusual morphology and a unique phenotype and remain attached to the culture flasks after trypsin digestion. Attempts to expand MPCs failed and revealed their “resting” state, which was also confirmed by Ki-67 negativity and evaluated by incorporation of 5-bromodeoxyuridine. Interestingly, when cultured in foetal calf serum (FBS) or human cord blood serum, MPCs were able to differentiate into highly proliferative and clonogenic MSCs. In parallel, MPCs were able to efficiently differentiate towards endothelial lineage, producing mature endothelial cells able to give rise to tube-like structures in 3D-cultures [19]. Moreover, some evidence suggests a cardiomyogenic differentiation capacity of MPCs, revealing the capacity to generate quadri- or trigonal cells positive for α-actinin and troponin I (data not shown). Taken together these results define MPCs as multipotent progenitors of mesodermal lineage, easily harvested from the bone marrow of adults. From the first characterization, MPCs revealed expression of the pluripotency-associated markers SSEA-4 and nuclear factors *Oct-4* and *Nanog*. Clarifying mechanism of *Oct-4* expression by these adult cells, and its regulation circuit, could lead to a better understanding of MPCs' biology and their clinical role. Furthermore, extended studies could reveal new interesting involvements for the transcription factor *Oct-4*. Here we present an extensive molecular characterization of highly purified MPCs, including 39 genes involved in stem cell machinery, differentiation and cell cycle regulation.

## Methods

### Ethical statement

The study protocol was approved by the ethical committees of Azienda Ospedaliera Universitaria Pisana. The fundamental principles of ethics in research on human participants were maintained throughout the study period. The research procedures were disclosed to all participants and written informed consent was obtained for sample collection.

### Donors and cell cultures from bone marrow

Bone marrow mononuclear cells were collected, after written consent, from twelve patients (9M/3F, median age: 61y) undergoing cardiac surgery. Cells were cultured using a standard protocol (Dulbecco's modified minimal essential medium, DMEM supplemented with 10% of FBS) to obtain MSCs, in parallel 800,000 cells/cm^2^ were cultured in DMEM supplemented with 10% of pooled human AB type serum (PhABS, Lonza; Walkersville MD-USA) in hydrophobic plastic flasks as previously reported [19]. After 12-15 days of culture cells were detached by TrypLE Select® (Invitrogen, San Diego CA-USA) digestion and gentle scraping. To assay purity of cultures, aliquots of cells were processed for cytofluorimetric analysis using antibodies for SSEA-4 AlexaFluor-488® conjugated (Biolegend, San Diego CA-USA), MSCA-1 PE-conjugated (Miltenyi, Bergisch Gladbach, GER), CD105 PE-conjugated (Biolegend) and CD90 PE/Cy5-conjugated (Biolegend). Data were acquired and analyzed by FACScan® equipped with CellQuest® Software (BectonDickinson, San Jose, CA-USA). MPC cultures were assessed and subsequently processed for molecular characterization when rates of MSCA-1^+^CD90^bright^ (MSC phenotype) were lower than 2%. Similarly, MSC cultures were processed if SSEA-4^+^MSCA-1^neg^CD90^neg^ (MPC phenotype) elements were <2%.

### Quantitative RT-PCR

Total RNA was extracted using an RNeasy Mini Kit (Qiagen GmbH, Hilden, Germany) as indicated by the manufacturer's protocol. On-column DNase I digestion was performed. 100 ng of each RNA sample was retrotranscribed with QuantiTect Whole Transcriptome Kit (Qiagen) and a 50-fold dilution of cDNAs was analyzed by quantitative Real Time PCR (qRT-PCR) using an iCycler-iQ5 Optical System (Bio-Rad Laboratories, Hercules, CA-USA) utilizing iQ SYBR Green SuperMix (Bio-Rad). All samples were run in duplicate. Primers (see Supplemental Table S1) were designed from coding sequences published in Gene Bank with the help of either Beacon Designer Software (Premier Biosoft International,Palo Alto, CA-USA) or from a Human Pluripotent Stem Cell Assessment Kit (R&D Systems, Minneapolis MN-USA) and clustered in four groups: “Pluripotency-associated genes” (including OCT4, its cofactors and target genes), “Commitment-associated genes”, “Sox family genes” and “other genes” of interest. Relative quantitative analysis was performed following 2-^ΔΔCt^ Livak method [21]. Normalization was performed with the housekeeping genes GAPDH and HPRT. Statistical analysis was performed by applying the two tailed *t* test. Subsequently, qRT-PCR of pluripotency-associated genes, SOX2 and SOX15 were also performed on mRNA extracted from three human embryonic stem cell (hESCs) lines; BG01V, I6 and H9. Inter-run calibration was performed by the internal calibrator method as previously described [22]. Telomere length assay was performed using the TeloTAGGG assay kit (Roche, Madison, WI-USA) according to the manufacturer's instructions, and median telomere restriction fragment (TRF) length was evaluated by Leica QWin image analysis software (Leica, Wetzlar, Germany).

### Tri-colors Immunofluorescence

FBS and PhABS cultures were performed, in parallel, on Permanox® double-chamber slides (Nunc, Rochester, NY-USA) as described above. After 8–10 days slides were fixed for 15 min in periodate-lysine-paraformaldheyde and made permeable by Triton-X100 0.05% for 30 min. Immunofluorescence was performed using anti-Oct-4 (Santa Cruz, CA-USA), anti-Nanog (BectonDickinson), anti-Sox15 and anti-Nestin (ABCam, Cambridge, UK) as primary antibodies and revealed by Goat anti-mouse SFX kit (Invitrogen) according to the manufacturer's instructions using AlexaFluor®-488 anti-mouse IgG. Subsequently slides were stained by Phalloidin AlexaFluor®-555 conjugated (Invitrogen) for 30 min to reveal actin organization, and mounted in Prolong® Gold antifade reagent with 4′,6-diamidino-2-phenylindole (DAPI) (Invitrogen) to allow nucleus localization. Pictures were taken and combined using a standard fluorescence DMR Leica microscope (Leica) equipped with Leica CW4000 image software (Leica).

### Dot-Blot Analysis

For three of the most abundant samples an aliquot of 300,000–600,000 cells from FBS or PhABS cultures was washed and pellets were processed for protein extraction of nuclei contents using the kit from Active-Motif (Carlsbad, CA-USA). Extracted proteins (10 µl) were spotted, in quintuplicate, on nitrocellulose membranes (Bio-Rad, Hercules CA-USA). Membranes were processed for staining with anti-Oct-4 (Santa Cruz) and anti-Sox15 (ABCam) and revealed by HRP-conjugated secondary antibody (ABCam) and subsequent incubation with ECL reagent (ABCam). Images were acquired by ChemiDoc digital imaging system (Bio-Rad) and evaluated by Leica Qwin Image Analysis Software (Leica). The data is presented as mean ± standard error (SE).

## Results

**Figure 1:**
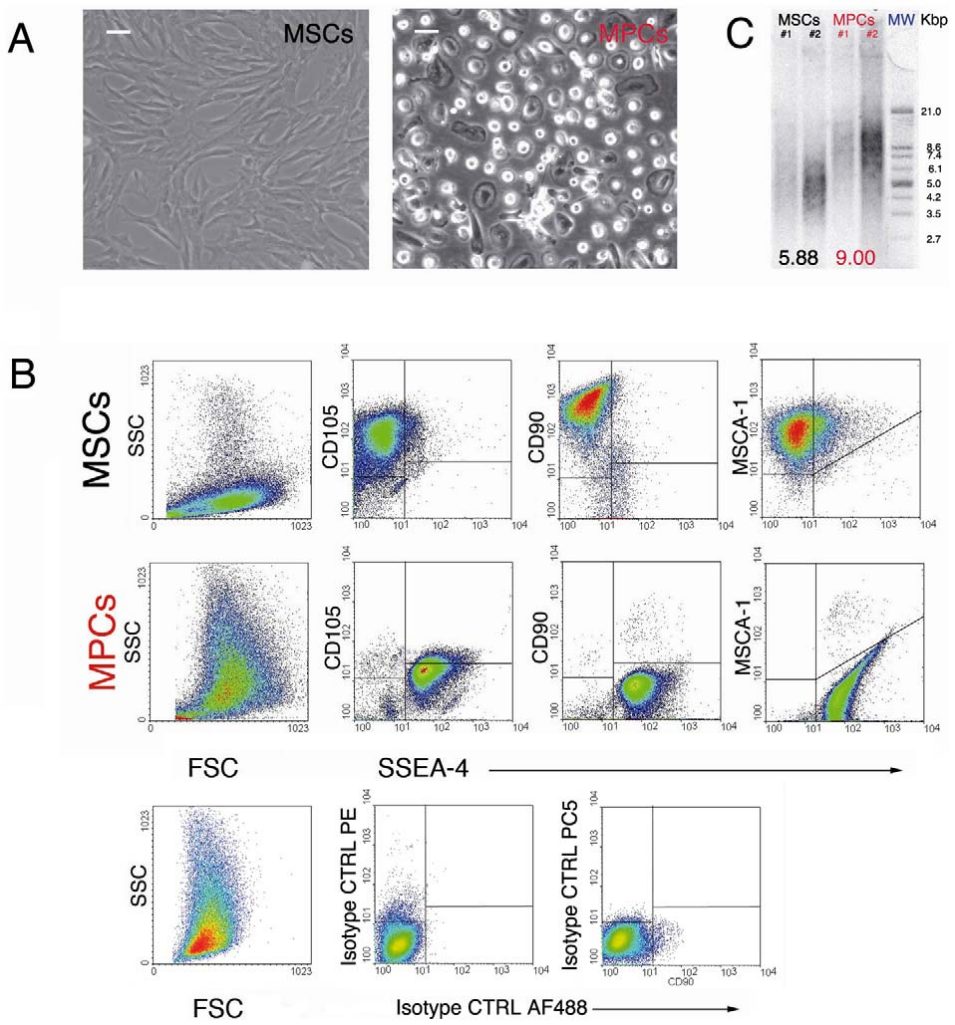
Mesodermal Progenitor Cells (MPCs) differ from MSCs and show unusual immature gene expression pattern. A) Mesodermal progenitor cells (MPCs) are easily distinguishable in culture from typical spindle-shaped MSCs by their characteristic fried egg-like shape (bars = 50 µm): rounded cells with a thick core region that is highly rifrangent. B) Cytofluorimetric analysis allows evaluation of the purity of selective cultures. MPCs show a high SSC signal, stain brightly with anti-SSEA-4 antibody and lack the mesenchymal markers CD90 and MSCA-1. MPCs share expression of CD105 with MSCs, but at a lower signal. C) Telomere length assay, values of medians TRF are reported, at the end of the lanes, for two paired samples (#1, #2, MW: molecular weight).

Ten samples produced highly monomorphic cultures (Figure 1A) showing typical spindle-shaped cells (MSCs) in FBS cultures, or rounded and highly rifrangent cells (MPCs) in PhABS cultures. Flow cytometry confirmed purity and phenotype of the cells (Figure 1B). As expected, MSCs were SSC^low^SSEA-4^neg^CD105^bright^MSCA-1^+^CD90^bright^, while MPCs were SSC^high^SSEA-4^+^CD105^dim^MSCA-1^neg^CD90^neg^. Two samples were rejected due to a higher contaminating MSC rate in MPC cultures. We also assessed telomere length utilizing the Southern method, determining median terminal restriction fragment (TRF) length. TRF length was shorter (5.88 kb) in MSCs than in MPCs (9.00 kb) (Figure 1C). This latter value is near to the values reported for passaged human embryonic cell lines [23] or human iPSCs [24].

**Figure 2:**
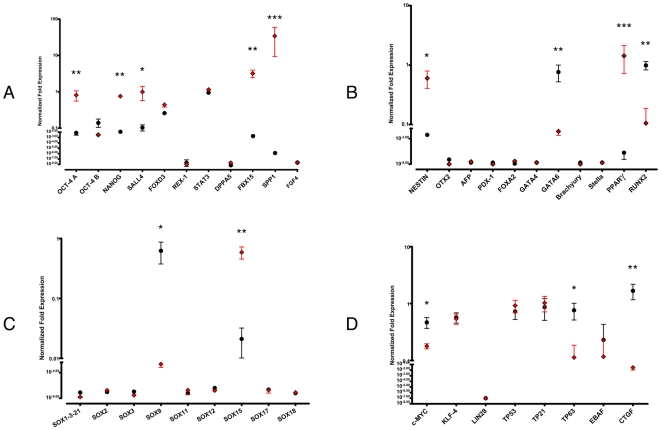
Quantitative RT-PCR analysis of 10 samples from MPCs (red squares) or MSCs (black dots). A) Pluripotency-associated genes, B) Commitment-associated genes, C) Sox family genes and D) other genes of interest. Data are normalized and expressed as media +/− SEM (* p<0.05; ** p<0.01, *** p<0.001).

Total mRNA was extracted, reverse transcripted and evaluated by quantitative RT-PCR for expression of genes involved in the characterization of stem cells: undifferentiated ESCs (OCT4, NANOG, SALL4, DPPA5, REX-1, STAT3, FBX15, FOXD3, SPP1, FGF4) (Figure 2A), ectodermal committed SCs (NESTIN, OTX2), endodermal committed SCs (α-Fetoprotein AFP, GATA-4, PDX-1, FOXA2), mesodermal committed SCs (Brachyury, GATA6, RUNX2, PPARγ) and germ cells (Stella) were evaluated (Figure 2B). Sox family genes were also evaluated (SOX1, SOX2, SOX3, SOX9, SOX11, SOX12, SOX15, SOX17, SOX18 and SOX21) (Figure 2C) alongside other reprogramming factors that included c-MYC, KLF4 and LIN28; *p53* circuit was evaluated by qRT-PCR for TP53, TP63 and TP21. To complete the analysis qRT-PCR was also performed to study two other genes regulated by *Oct-4/Sox15* complex i.e. CTGF and EBAF (Figure 2D).

**Figure 3:**
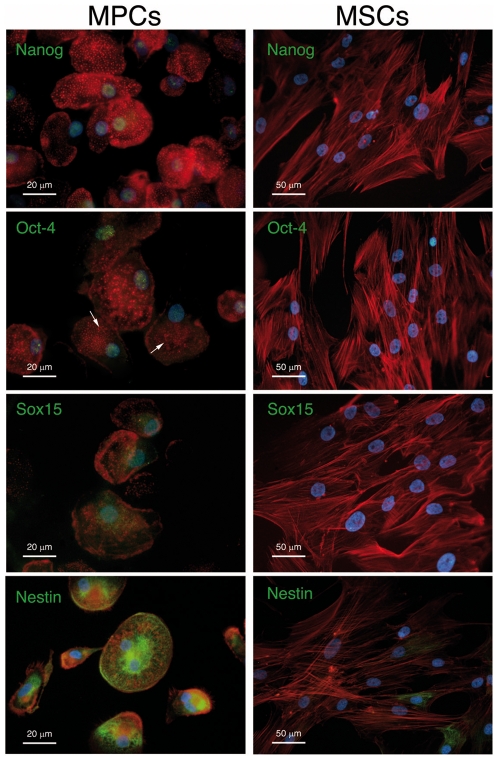
MPCs express *Nanog*, *Oct-4* and *Sox15* but no *Sox2* in the nucleus. Indirect immunofluorescent stain confirms the expression of *Nanog*, *Oct-4* and *Sox15* (green) in MPCs' nuclei (blue) but not in MSCs easily distinguishable from mesodermal progenitors by different spatial organization of F-actin (red). MPCs also differ from MSCs due to their unexpected high expression of well-organized Nestin filaments (green) as shown by the lower panels. Green fluorescent isotype controls reveal no specific signal (data not shown).

Quantitative RT-PCR analysis revealed high levels of genuine OCT-4 (isoform A) mRNA in MPCs only (p<0.01) alongside the expression of NANOG (p<0.01) (Figure 2A). FOXD3, STAT3, SALL4 usually involved in OCT4 and NANOG activation in pluripotent stem cells were also detected in MPCs. *Oct-4* target genes such as REX-1 and DPPA5 or FGF4 were not detected. In contrast, consistent expression of FBX15 (p<0.01) and SPP1 (p<0.001) was revealed in MPCs. None of the commitment-associated genes were detected except NESTIN (p<0.05) and PPARγ (p<0.001), which were highly expressed by MPCs (Figure 2B). Despite these cells expressing the above mentioned genes, typically associated with *Oct-4* circuit activation, SOX2 expression was not revealed in MPCs, while high levels of SOX15 (p<0.01) were detected (Figure 2C), suggesting the possibility of direct interaction of *Oct-4* and *Sox15* (in place of *Sox2*). Expression of MPC exclusively associated markers (*Nanog*, *Oct-4*, *Sox15* and *Nestin*) was confirmed by immunofluorescence (Figure 3). Actin organization allows the identification of MPCs. As previously reported [18], [19], MPCs showed a dotted pattern of F-actin, often organized in rosettes (arrows). In contrast, long fibres of polymerized actin characterized MSCs. Nuclear localization of *Oct-4* and *Sox15* was further confirmed by dot-blot analysis of nuclear proteins extract (Supplemental Figure S1). In the “other genes” panel we reported expression of KLF4, c-MYC but not LIN28. TP53 and TP21 were expressed, with no significant differences, by the two populations, but otherwise TP63 levels were lower in MPCs (p<0.05) (Figure 2D).

**Figure 4:**
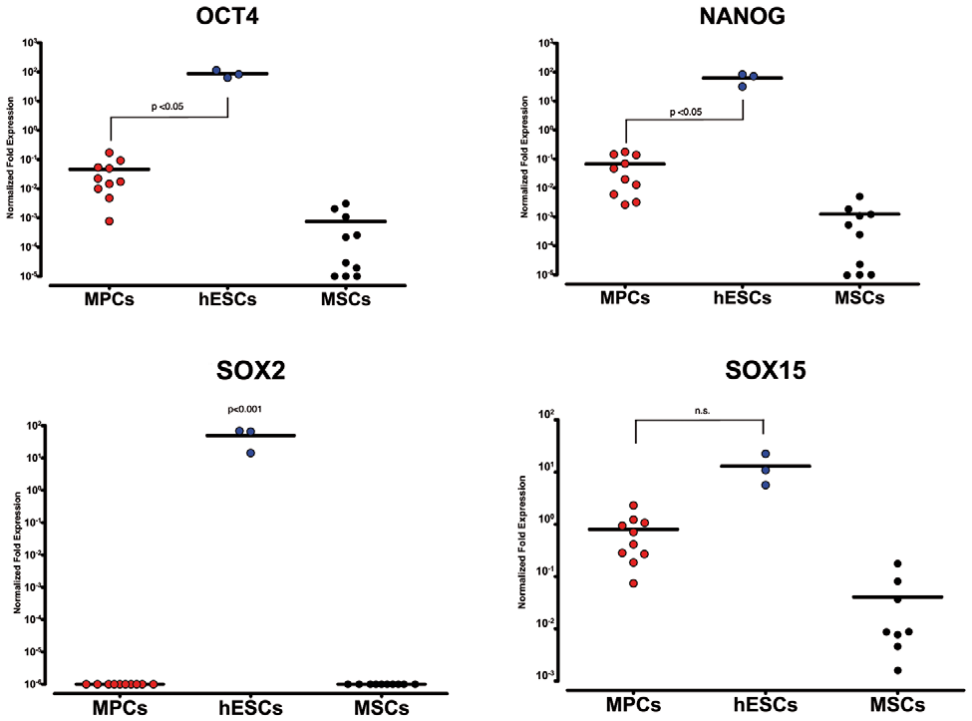
Comparison of median relative normalized expression of OCT4, NANOG, SOX2 and SOX15 among MPCs, MSCs and hESC lines. qRT-PCR data from MPC (red dots) and MSC (black dots) populations were calibrated to be compared to hESC signals (blue dots).

To compare the *Oct-4* “adult” circuit in MPCs to the embryonic one, we performed qRT-PCR of pluripotency-associated genes, SOX2 and SOX15 on mRNA from three human embryonic cell lines (BG01V, I6 and H9). As might be expected, median relative normalized expression of OCT4 and NANOG in the embryonic lines turned out to be 2.5 logs higher (p<0.05) than in MPCs (Figure 4). As expected, hESC lines showed consistent expression of SOX2 and SOX15 was also expressed with no significant difference compared to MPCs. Some *Oct-4* target genes/co-factors not expressed by MPCs, such as REX-1, DPPA5 and FGF4, were in contrast expressed in hESCs while SALL4 and FOXD3 showed increased expression in embryonic cells (p<0.01, data not shown). Despite these quantitative differences in OCT4 and SOX2 expression, some *Oct-4* target genes, such as KLF4, FBX15 and SPP1, were expressed with no significant differences between MPCs and hESCs (data not shown).

## Discussion

Our results define MPC as a human progenitor easily obtainable from adult bone marrow that constitutively express *Oct-4*, which is considered to be the core factor associated with pluripotency. From the first characterization and isolation in adult bone marrow, MPCs reveal their early progenitor nature, together with their ability to produce several generations of MSCs and different multilineage commitments [19]. We initially reported expression of *Oct-4* and *Nanog* by immunofluorescence and qualitative RT-PCR, so that we hypothesized that MPCs could show a molecular program regulated by *Oct-4*. The results presented in this paper confirm the genuine expression of functional *Oct-4*, avoiding the misinterpretation due to pseudogenes and non-functional isoforms. Importantly, only the long isoform, OCT4A, has been reported to be responsible for the stem properties [25] and can sustain the stem cell self-renewal [26].

Extensively observed in embryonic stem cells, MPCs reveal unusual activation of the *Oct-4* regulatory circuit probably related to the lack of *Sox2*. The deregulation of “embryonic” *Oct-4/Sox2* target genes (FGF4, FBX15, and OCT4, SOX2 themselves) suggests the activity of the complex *Oct-4/Sox15* (in place of *Oct-4/Sox2* which is the core transcription complex in the “embryonic” circuit or “canonical” circuit). Detection of SOX15 in MPCs in a quantity similar to hESC could explain the expression of FBX15 that is reported as being also an *Oct-4/Sox15* target gene in murine ESCs [27]. On the contrary, CTGF, OTX2 and EBAF were not up-regulated in MPCs, in contrast to the reported data on hESCs, suggesting a defective activity of complex *Oct-4/Sox15* or the activation of a further control mechanism for gene transcription. This possibility is suggested by consistent expression of SPP1 in MPCs. In fact, Botquin *et al.* reported that SPP1 reveals a palindrome *Oct* factor recognition element (PORE) in the enhancer region, which is able to bind homodimers of POU proteins. The authors provided consistent evidence that the PORE region of SPP1 is a target for the homodimer of *Oct-4* and that this binding transactivates more strongly than the canonical octamer motif [28]. As reported, *Spp1* was secreted by pre-implantation embryos and hESCs lines express SPP1 at different levels. Interestingly, different levels of SPP1 expression inversely correlates with the expression levels of *Sox2*, which inhibit the *Oct-4* homodimer activity.

It is possible that adult *Oct-4* activity, detected in MPCs, involve interaction with *Sox15* (binding HMG domains) alongside self-dimerization (binding POREs), resulting in a characteristic transcriptional signature. In “adult” *Oct-4* circuit the SOX2 gene is fully silenced while the other pluripotency-associated co-factors such as NANOG, KLF4, cMYC and SALL4 are expressed with insignificant differences in MPCs and hESCs. The lack of SOX2 seems to represent the principal difference between pluripotent and adult *Oct-4* circuits, it could play a very important role in control of the MPC's fate and may be an important difference in their ontogenesis.

Taken together, these results suggest that the complex *Oct-4/Sox2* is the core factor which differentiates the pluripotent (embryonic) *Oct-4* molecular program from the adult one detected in *Oct-4* expressing MPCs. Further confirmation is provided by recent papers reporting that a fully pluripotent state can be induced in *Sox*2 expressing neural stem cells in mice [16] and in humans [17] by single factor *Oct-4* transfection. This suggests that the sole induction of *Sox2* in MPCs could lead to trigger a reprogramming process in easily obtainable bone marrow cells. The constitutive expression of co-factors like *Nanog*, *Klf-4* and *c-myc* in MPCs further sustains the single factor (1F) reprogramming hypothesis, as reported for foetal neural induced stem cells. *Klf-4* and *c-myc* endogenously expressed in the starting cells, make forcing their expression dispensable [17].

Moreover, efficiency in reprogramming MPCs could be expected to be increased when compared to somatic cells due to their early undifferentiated nature. In fact, it has been reported that adult progenitors are more reliable for reprogramming than terminally differentiated cells [14], [15] due to the less stringent epigenetic restriction that favours nuclear reprogramming. Nevertheless, the epigenetic restriction is only one of the conditions that limit the kinetics and efficiency of pluripontency induction. In fact, forcing expression of *Oct-4*, *Sox2*, *Klf-4* and *c-myc* triggers senescence and up-regulation of *p53/p21* [29], which negatively influence the percentage of fully reprogrammed iPSCs. In MPCs, high expression of *Oct-4* and *Nanog* and other co-factors is not associated with a significantly increased expression of *p53* and *p21*, compared to proliferating hESC or MSCs, alongside long telomeres.

The true significance for the adult *Oct-4* circuit in “*in vivo*” precursor of MPCs is still unclear: it could be required for the maintenance of immature features together with the need to preserve bone marrow homeostasis, but it cannot be ruled out that its deregulation could be at the basis for transforming MPCs into tumour-initiating cells. Recently, it has been reported that the expression of *Oct-4*, and co-factors, plays a fundamental role in the induction of cancer stem cells, as tumour-initiating cells in Ewing's sarcoma [30].

MPCs are very attractive and could open new prospective clinical applications for reprogrammed cells. Further studies are needed to attempt reprogramming not only by SOX2 transfection but also by evaluating the opportunities to induce expression of *Sox2* by signalling modulation, i.e Wnts [31], Activin/Nodal [32] and/or Hedgehog [33].

Over the last few years, *in vitro* induction of pluripotency has seemed to offer greater potential when compared to the problematic isolation of rare adult stem cells in the potential use of pluripotent cells in therapy. However, the generation of an efficient virus-free strategy for reprogramming represents a crucial challenge that has to be considered in new approaches. MPCs could be an alternative, as they are not so rare in adult bone marrow and the isolating procedure is easy and inexpensive [18]. MPCs reveal a peculiar *Oct-4* circuit activation that, hopefully, could be modulated toward pluripotency without genetic manipulation, i.e. by signal inhibition as reported for NSCs that are presently unavailable in human adults.

## Supporting Information

Figure S1 Dot-Blot analysis of nuclear extract detect presence of Oct-4 and Sox15 transcription factors in MPCs. In three MPC samples with higher cell count it has been possible to perform dot-blot analysis to confirm the presence of Oct-4 and Sox15 in the nuclear proteins extracts. Densitometric evaluation of spots were performed excluding the higher and the lower signal of the five repeats.
(0.54 MB TIF)

Table S1 Primer pairs designed for gene expression analysis.
(0.42 MB RTF)
