# Supplementary material for: Constitutive Expression of Pluripotency-Associated Genes in Mesodermal Progenitor Cells (MPCs)
Source: PLoS One. 2010 Mar 25;5(3):e9861. doi: 10.1371/journal.pone.0009861 (PMC2845604; doi:10.1371/journal.pone.0009861)
Supplement: Table S1 — Primer pairs designed for gene expression analysis. (0.42 MB RTF) [file pone.0009861.s002.rtf]

NCBI Ref#	Gene Name	Primer Pair	Amplicon	
			length	
 	 		 	 	
NM_024865	NANOG	sense	GAACTCTCCAACATCCTGAACCTC	127 bp	
		anti-sense	CCTTCTGCGTCACACCATTGC		
 	 		 	 	
NM_002701	POU5.1 variant 1	sense	CCGTGAAGCTGGAGAAGGAGAAG	198 bp	
		anti-sense	AGCGGCAGATGGTCGTTTGG		
 	 		 	 	
NM_203289	POU5.1 variant 2	sense	ACTGGTGTGTTTATGTTCTTAC	192 bp	
		anti-sense	AGCGGCAGATGGTCGTTTGG		
 	 		 	 	
NM_006617	NESTIN	sense	GTTGGAACAGAGGTTGGAG	250 bp	
		anti-sense	GAGGGAAGTCTTGGAGCC		
 	 		 	 	
NM_005986, NM_005634, 	SOX1-3-21	sense	GCCTTCATGGTGTGGTCC	221 bp	
NM_007084		anti-sense	AGCAGCGTCTTGGTCTTG		
 	 		 	 	
NM_003106	SOX2	sense	GTTGTCAAGGCAGAGAAGAG	228 bp	
		anti-sense	GAGAGAGGCAAACTGGAATC		
 	 		 	 	
NM_005634	SOX3	sense	CCATCGCATCGCACTCTC	174 bp	
		anti-sense	GCACCGTTCCGTTGACTG		
 	 		 	 	
NM_000346	SOX9	sense	GGTCAGCCAGGTGCTCAAAG	250 bp	
		anti-sense	CTCCGCCTCCTCCACGAAG		
 	 		 	 	
NM_003108	SOX11	sense	GCTGCTGAGACGCTACAAC	171 bp	
		anti-sense	TGCTGCTTGGTGATGTTCTTG		
 	 		 	 	
NM_006943	SOX12	sense	AGGAGTCGCTGGGCTTTC	213 bp	
		anti-sense	GACGGTGGGCTCAGTAGG		
 	 		 	 	
NM_006942	SOX15	sense	TGCTGCCTCCTCATCTTCG	226 bp	
		anti-sense	CCGCTTCTCGTCCTCGTC		
 	 		 	 	
NM_022454	SOX17*		 	194 bp	
			 		
 	 		 	 	
NM_018419	SOX18	sense	GCCCAGAGGAGAGCAGCC	173 bp	
		anti-sense	GCGTATGAGAGAGCAGAGCG		
 	 		 	 	
NM_021784	FoxA2	sense	CCAGCAGAGCCCCAACAAGATG	234 bp	
		anti-sense	CGCAGGTAGCAGCCGTTCTC		
 	 		 	 	
NM_012183	FoxD3	sense	CAGCGGTTCGGCGGGAGG	218 bp	
		anti-sense	TGAGTGAGAGGTTGTGGCGGATG		
 	 		 	 	
NM_174900	REX-1	sense	GAGGTGGCATTGGAAATAGCAGAG	177 bp	
		anti-sense	CAAGGGAGGCGGTGAGTGG		
 	 		 	 	
NM_004235	KLF-4	sense	CAGAGGAGCCCAAGCCAAAGAG	217 bp	
		anti-sense	CGGTAGTGCCTGGTCAGTTCATC		
 	 		 	 	
NM_002467	c-MYC	sense	CCCGCTTCTCTGAAAGGCTCTC	198 bp	
		anti-sense	CTCTGCTGCTGCTGCTGGTAG		
 	 		 	 	
NM_020436	SALL-4	sense	GCCGCACTGAGATGGAAGGTC	231 bp	
		anti-sense	GAGTCCGCTCGTGGATCTGAAG		
 	 		 	 	
NM_024674	LIN28	sense	CGGGCATCTGTAAGTGGTTCAAC	249 bp	
		anti-sense	GCCGCCTCTCACTCCCAATAC		
 	 		 	 	
NM_002052	GATA-4	sense	CTACTCCAGCCCCTACCC	200 bp	
		anti-sense	ACATAGCCCCACAGTTGAC		
 	 		 	 	
NM_005257	GATA-6	sense	CACAACACAACCTACAGCCTCAG	107 bp	
		anti-sense	GCCCATCTTGACCCGAATACTTG		
 	 		 	 	
NM_003722	TP63	sense	AAGAACGGTGATGGTACGAAG	270 bp	
		anti-sense	TGAAGATGGAGACTGTATTGAGG		
 	 		 	 	
NM_000546	TP53	sense	TCAACAAGATGTTTTGCCAACTG	118 bp	
		anti-sense	ATGTGCTGTGACTGCTTGTAGATG		
 	 		 	 	
NM_078467	TP21	sense	GCCCGTGAGCGATGGAAC	175 bp	
		anti-sense	GCAGCAGAGCAGGTGAGG		
 	 		 	 	
NM_001901	CTGF	sense	AGCGGAGAGTCCTTCCAGAGC	228 bp	
		anti-sense	CGTGTCTTCCAGTCGGTAAGCC		
 	 		 	 	
NM_003240	EBAF	sense	AAGGCTTTGGCTCGTCATTTCC	129 bp	
		anti-sense	CGGGCAAGGCTCAGTCTCC		
 	 		 	 	
NM_003240	OTX2	sense	GCCGCCAACAACAGCAACAACAG	233 bp	
		anti-sense	GCAGGAAGAGGAGGTGGACAAGGG		
 	 		 	 	
NM_152676	FBX15	sense	GCCTAGCATCATTGTCAACCTTAG	226 bp	
		anti-sense	GCCAGTTCTTCCTCCTTCTTCC		
 	 		 	 	
NM_213662	STAT-3	sense	ACCAAGCGAGGACTGAGCATC	148 bp	
		anti-sense	GCCAGACCCAGAAGGAGAAGC		
 	 		 	 	
NM_001025290	DPPA5*		 	484 bp	
			 		
 	 		 	 	
NM_001134	α-Fetoprotein*		 	537 bp	
			 		
 	 		 	 	
NM_003181	Brachyury*		 	430 bp	
			 		
 	 		 	 	
NM_000209	PDX-1*		 	262 bp	
			 		
 	 		 	 	
NM_022454	Stella*		 	445 bp	
			 		
 	 		 	 	
NM_001040060	SPP1	sense	GCCGAGGTGATAGTGTGGTT	101 bp	
		anti-sense	TGAGGTGATGTCCTCGTCTG		
 	 		 	 	
NM_004348	RUNX2	sense	CCAACCCACGAATGCACTATC	91 bp	
		anti-sense	TAGTGAGTGGTGGCGGACATAC		
 	 		 	 	
NM_015869	PPARγ	sense	GATACACTGTCTGCAAACATATCACAA	91 bp	
		anti-sense	CCACGGAGCTGATCCCAA		
 	 		 	 	
NM_001101	β-actin	sense	CGCCGCCAGCTCACCATG	120 bp	
		anti-sense	CACGATGGAGGGGAAGACGG		
 	 		 	 	
NM_004048	β2-microglobulin	sense	GAGTATGCCTGCCGTGTG	110bp	
		anti-sense	AATCCAAATGCGGCATCT		
 	 		 	 	
NM_000194	HPRT	sense	AGACTTTGCTTTCCTTGGTCAGG	101 bp	
		anti-sense	GTCTGGCTTATATCCAACACTTCG		
 	 		 	 	
NM_002046	GAPDH	sense	CCCTTCATTGACCTCAACTACATG	115 bp	
		anti-sense	TGGGATTTCCATTGATGACAAGC		
 	 		 	 	
NM_000988	RPL27	sense	ATCGCCCCTACAGCCATG	200 bp	
		anti-sense	GAAGACATCCTTATTGACGACAG		
 (*) From Human Pluripotent Stem Cell Assessment Kit (R&D Systems, Minneapolis-MN USA)
